# Mr. William Stevenson Clarke in Answer to Chirurgicus

**Published:** 1817-01

**Authors:** 


*For the London Medical and Physical Journal.*

### Mr. William Stevenson Clarke *in Answer to Chirurgicus.*

HAVING read, in your Journal for November, a *most potent eulogium* upon what the author is pleased to call an eulogium of mine, on the effects of general depletion, I have thought it right to make a few observations upon some of the principal passages, although it may be considered unnecessary by many of your readers, particularly as the production of an anonymous correspondent. Chirurgicus commences (after a short but pious lucubration) with a quotation from my paper—" It seems that from the 24th of June until the 3d of July, not less than 111 ounces of blood had been ordered to be drawn from the system, to affect a part exterior to the body, and not essentially necessary to the functions of life." Chirurgicus adds, " It then occurred to him, after observing no good effect whatever on this exterior part, from the loss of 95 ounces of blood from the system, to call to his aid those useful little animals—leeches, to the number of ten, and that, on the day after their use, his patient was not worse, nor was the testicle much increased in size,—a circumstance which," he says, " by the bye, did take place after every previous bleeding from the system." If he will read the paper in question with even the most superficial attention, he will discover that very considerable relief was obtained after the bleeding on the 30th of June; that this relief continued without intermission till the morning of the 3d of July, at which time a " considerable increase of the pain and inflammation came on."

Chirurgicus then proceeds—" Yet, Mr. Editor, he has not calculated on the remote effects on the constitution of his patient, by that wasteful plan with which he had commenced and continued the treatment of this very common disease."

This

This is also an error. I had, with the most scrupulous attention, calculated upon both the present and future effects on the constitution of my patient. I had to deal with a robust, plethoric, and highly-inflamed system, with an organ immensely inflamed and swelled,—that inflammation rapidly spreading up the chord, and affecting the peritonæal lining, to a certain extent, within the cavity; and, superadded to all this, high constitutional irritation;—in fact, with every circumstance that appeared to threaten a speedy destruction of the texture of the part. Under such circumstances, was it the duty of an active practitioner to trifle with the case, by the application of a few leeches?—certainly not. I did not, therefore, hesitate to adopt the "wasteful plan," with evident advantage at the time; and, in respect to its after-effects on the constitution, I can also satisfactorily calm the fears of Chirurgicus, by informing him that I have lately seen the patient several times, perfectly restored to health and strength, and with that *trifling* organ, the testis, free from disease, which, to all appearance, would not have been the case, had the *active plan* recommended by Chirurgicus been followed.

Chirurgicus then proceeds—" If a residence within the Lock Hospital, a situation where Mr. Clark must have seen this disease, has not already taught him this lesson, I beg that he will take it from *this*, his case, viz. that local evacuations are infinitely better than exhausting the system by such large bleedings from the system, a practice which, I regret to observe, making a most fearless progress, regardless of the ulterior effects on the constitution." If the practice within the Lock Hospital has not taught me this lesson, I can assure Chirurgicus that the luminous advice contained in his paper will not; nor do I think it will have much influence on the minds of the majority of practitioners, either in this or other countries, whose pens are constantly employed in paying their tribute of respect to that important practice, by laying before the public their experience of the successful termination of many hitherto fatal diseases, from a well-directed and scientific system of depletion.

None there are in this country, I trust, who would sacrifice even the most wretched of their fellow mortals, in order to gratify their propensity for any favourite theory, or even "wantonly" to carry the most approved mode of treatment to an extreme, so as to endanger the lives of their patients, —an assertion so unwarrantably advanced by the anonymous author of that paper.

I shall close the controversy with Chirurgicus by noticing the last paragraph in my adversary's paper:—" I am not,"

 says says he, "a young man; I have lived to see the effects of the inconsiderate use of this potent instrument, and I have great consolation in reflecting that I have never used it *wantonly*."

Every surgeon of candour will believe that the lancet is never used *wantonly;* but the cautious language of Chirurgicus may lead the most experienced practitioners to fear that his patients may often have suffered by his caution or timidity.

*York; Nov.* 25, 1816.

---

*For the London Medical and Physical Journal.*

